# Wenyang Zhenshuai Granules inhibits cardiomyocyte apoptosis in chronic heart failure by regulating p38 MAPK signaling pathway through exosomal miR-155

**DOI:** 10.3389/fphar.2025.1538091

**Published:** 2025-10-03

**Authors:** Liqi Peng, Xinyu Chen, Huzhi Cai, Yanping Tang, Qingyang Chen, Fang Zhou

**Affiliations:** ^1^ The First Clinical College of Traditional Chinese Medicine, Hunan University of Chinese Medicine, Changsha, China; ^2^ Preventive Treatment Center, The First Affiliated Hospital of Hunan University of Chinese Medicine, Changsha, China; ^3^ International Medical Department, The First Affiliated Hospital of Hunan University of Chinese Medicine, Changsha, China; ^4^ College of Integrative Medicine, Hunan University of Chinese Medicine, Changsha, China; ^5^ Intensive Care Unit, The First Affiliated Hospital of Hunan University of Chinese Medicine, Changsha, China; ^6^ Health Management Department, The First Affiliated Hospital of Hunan University of Chinese Medicine, Changsha, China

**Keywords:** chronic heart failure, exosome, miR-155, p38 MAPK, cardiomyocyte apoptosis, doxorubicin, Wenyang Zhenshuai Granules

## Abstract

**Background:**

Chronic heart failure (CHF) represents a significant global public health concern, warranting further investigation and intervention. Wenyang Zhenshuai Granules (WZG) is an in-hospital preparation of the First Affiliated Hospital of Hunan University of Chinese Medicine, which has been approved by the Hunan Provincial Drug Administration (Approval No.: Z20190105000) for the treatment of CHF. The objective of this study was to examine the impact of WZG on cardiomyocyte apoptosis in CHF through the regulation of the p38 mitogen-activated protein kinase (MAPK) signaling pathway by exosomal microRNA-155.

**Methods:**

Doxorubicin (DOX) was employed to construct a model of cardiomyocyte injury associated with CHF. The H9c2 cells were divided into four groups: the normal control group (NC), the DOX group (DOX), the DOX + drug-containing serum group (DOX+WZG), and the DOX + enalapril (ENP) group (DOX+ENP). The morphology of the cardiomyocytes was observed at 15, 30, and 45 h into the experiment using an inverted microscope. The viability of cells and the number of apoptotic cells were determined through the use of a CCK-8 assay and flow cytometry, respectively. Subsequently, exosomes were extracted and subjected to morphological characterization and identification. The expression of exosomal miR-155, the p38 MAPK signaling pathway, and apoptotic proteins were examined.

**Results:**

The results demonstrated that WZG could enhance the morphology of H9c2 cells, diminish the apoptosis rate of cells, and augment the viability of cells. Western blot and RT-qPCR assays provided further confirmation that WZG could promote the secretion of exosomes from cardiomyocytes, increase the content of miR-155 in exosomes, and inhibit the activation of the p38 MAPK signaling pathway.

**Conclusion:**

WZG inhibits p38 MAPK protein phosphorylation via exosomal miR-155, thereby exerting anti-apoptotic effects on cardiomyocytes in CHF.

## 1 Introduction

Chronic heart failure (CHF) is a complex group of syndromes in which ventricular filling or ejection capacity is impaired, and is clinically characterized by dyspnea, reduced exercise tolerance, and edema (Roger, 2021). The prevalence of CHF has reached a level of global public health concern, with the condition imposing a significant medical burden on society. Statistical data from the American Heart Association indicate that there are currently 6 million patients with CHF, representing approximately 1.8% of the total U.S. population (Virani et al., 2021). Based on domestic epidemiological surveys, the prevalence of CHF has been estimated at 0.9%, with an estimated 8.9 million patients (Metra et al., 2023). It is projected that by 2030, the number of individuals with CHF in China will reach 23.3 million (The, 2023). Despite the efficacy of traditional and novel drug therapy in improving the prognosis of patients with CHF, adverse drug reactions and poor patient compliance remain significant challenges.

Exosomes are microvesicles with diameters of 40–100 nm derived from endocytosis and are widely distributed in biological fluids including blood, urine, bile, saliva, etc. (Kalluri and LeBleu, 2020) Exosomes serve as crucial mediators of intercellular communication. An expanding body of evidence from scientific research indicated that exosomes play a pivotal role in the pathogenesis of numerous cardiovascular diseases (CVDs) by transporting microRNA (Han et al., 2022; Saliminejad et al., 2019). In recent years, exosomal microRNA has emerged as a significant area of investigation in the pathogenesis of CHF, with considerable potential for the development of novel diagnostic and therapeutic tools (Meiri et al., 2020).

Among various microRNAs implicated in cardiovascular diseases, miR-155 has gained particular attention due to its critical role in the regulation of cardiomyocyte apoptosis. This is accomplished by binding to the non-coding region at the 3′ end of target mRNAs, which results in mRNA degradation or protein synthesis inhibition (Guo et al., 2023; Liu et al., 2024). It has been evidenced that miR-155 expression levels are frequently elevated in patients with CVDs. The downregulation of endogenous miR-155 expression has been shown to protect the heart from pathological myocardial hypertrophy (Eshraghi et al., 2024; Seok et al., 2014). Furthermore, it has been established that exosomes derived from M1 macrophages can inhibit cardiomyocyte proliferation by delivering miR-155 (He et al., 2024). The cardioprotective mechanism of exosomal miR-155 has recently emerged as a prominent area of investigation within the field of CVDs.

p38 mitogen-activated protein kinase (MAPK) is a pathway protease that has been associated with a number of biological processes, including apoptosis and inflammatory responses (Sanz-Ezquerro and Cuenda, 2021). p38 MAPK is markedly expressed in the human heart and has been elucidated as a pivotal factor in the development of CHF (Romero-Becerra et al., 2020). It has been established that the activation of p38 MAPK can result in the expression of genes associated with myocardial hypertrophy or apoptosis, thereby precipitating myocardial injury (Shati, 2020). Notably, recent findings suggest that exosomal miR-155 may influence inflammation and apoptosis through modulation of the p38 MAPK pathway (Yu et al., 2020). Specifically, miR-155 has been shown to directly target MAPK14 (encoding p38α), thereby suppressing its protein expression and inhibiting downstream signaling (Jia et al., 2020). In addition, miR-155-5p indirectly regulates p38 MAPK activation by targeting MSK1, which modulates the expression of DUSP1, a known inhibitor of p38 phosphorylation (Xu et al., 2023). These findings suggest both direct and indirect mechanisms through which miR-155 may influence the p38 MAPK pathway.

Given its role in regulating apoptosis and its enrichment in exosomes, miR-155 is increasingly recognized as a critical mediator in CHF pathophysiology via modulation of the p38 MAPK pathway. Our previous study demonstrated that Wenyang Zhenshuai Granules (WZG), a traditional Chinese medicine (TCM) formula, attenuated myocardial injury in rats with cardiorenal syndrome (CRS) by upregulating exosomal miR-155 and inhibiting p38 MAPK phosphorylation (O et al., 2020). In light of our previous findings and the extant published literature, we hypothesized that WZG may exert cardioprotective effects by modulating exosomal miR-155 and its downstream target, the p38 MAPK pathway.

The formulation of WZG consists of seven Chinese herbs, namely, *Aconitum carmichaelii Debeaux, Panax ginseng C.A.Mey., Zingiber officinale Roscoe, Poria cocos (Schw.)Wolf, Schisandra chinensis (Turcz.) Baill., Ophiopogon japonicus (Thunb.) Ker Gawl., and Glycyrrhiza uralensis Fisch. ex DC*. Clinical trials have provided evidence that WZG is an efficient TCM compound formula for the treatment of CHF. The formula has been demonstrated to enhance cardiac function, diminish serum NT-proBNP levels, and impede ventricular remodeling (VR) in patients with CHF (Lin et al., 2019). Prior work has indicated that WZG protects against doxorubicin (DOX)-induced cardiomyocyte injury via the LncRNA BIC/miR-155 axis and inhibition of MAPK signaling (Xu et al., 2020). Nevertheless, the precise mechanism by which WZG exerts its influence on cardiomyocyte apoptosis by regulating the p38 MAPK signaling pathway via exosomal miR-155 remains to be fully elucidated.

In this study, we employed DOX to develop an *in vitro* cardiomyocyte injury model, investigate the protective impact of WZG on cardiomyocytes, and examine the molecular mechanism through which WZG regulates the phosphorylation of p38 MAPK via exosomal miR-155. These findings provide experimental evidence to support the potential therapeutic utility of WZG in the treatment of CHF.

## 2 Methods and materials

### 2.1 Drugs and reagents

WZG was obtained from the First Affiliated Hospital of Hunan University of Chinese Medicine (Changsha, China). DOX hydrochloride (H44024359) was purchased from Shenzhen Main Luck Pharmaceuticals Inc. (Guangdong, China). Enalapril tablets (H32026567) were sourced from Yangzijiang Pharmaceutical Group Co., Ltd. (Jiangsu, China). Trizol reagent (15596026) was acquired from Thermo Fisher Scientific (Waltham, MA, USA). The mRNA reverse transcription kit (CW2569), miRNA reverse transcription kit (CW2141), UltraSYBR Mixture (CW2601), and DM2000 Plus DNA Marker (CW0632) were provided by Beijing Cowin Biotech Co., Ltd. (Beijing, China). The Annexin V-APC/PI apoptosis detection kit (KGA1030) was obtained from Jiangsu Keygen Biotech Corp., Ltd. (Jiangsu, China). The cell counting kit-8 (CCK-8) (NU679) was purchased from Dojindo (Kumamoto, Japan). The bicinchoninic acid (BCA) protein assay kit (P0012) was obtained from Beytotime Biotechnology (Shanghai, China). Antibodies against p38 MAPK (AWA48968), p-p38 MAPK (AWA48966), CD9 (AWA44344), CD63 (AWA43088), Goat anti-Mouse IgG (H+L) (AWS0001), and Goat anti-Rabbit IgG (H+L) (AWS0002) were acquired from Changsha Abiowell Biotechnology Co., Ltd. (Changsha, China). Antibodies against Caspase-3 (19677-1-AP) and *β*-actin (66008-1-Ig) were purchased from Proteintech Group (Rosemont, IL, USA). Antibodies against Bcl‐2 (ab196495) and Bax (ab32503) were provided by Abcam (Cambridge, MA, USA).

### 2.2 Preparation of WZG

The medicinal plants used in the composition of WZG (Table 1) were all procured from the First Affiliated Hospital of Hunan University of Chinese Medicine (Changsha, China). Each herb was rigorously authenticated by pharmacognosy expert Yang Lei. All plant materials were verified using the Medicinal Plant Names Services (MPNS) (http://mpns.kew.org/mpns-portal/) and Plants of the World Online (POWO) (http://www.plantsoftheworldonline.org). The herbs are washed, air-dried, and then weighed. *Panax ginseng C.A.Mey.* is boiled in water twice. The initial boiling is conducted for a duration of 2 h, while the subsequent boiling is carried out for a period of 1.5 h. The decoctions are subsequently combined, filtered, and the filtrate is set aside. The remaining six herbs are boiled in water on two separate occasions, with each boiling lasting for a period of 2 h. The decoctions are subsequently combined and filtered. The filtered liquid is combined with the *Panax ginseng C.A.Mey.* decoction, concentrated to a relative density of 1.20–1.25 at a temperature of 60 °C, and made into a thick paste. Subsequent to this step, the mixture undergoes a process of granulation, drying, and finally packaging.

**TABLE 1:** Composition of Wenyang Zhenshuai Granules.

Materials	TCM materials name	Latin name	Part used	Location
*Aconiti lateralis radix praeparata*	Heishunpian	*Aconitum carmichaelii Debeaux*	Lateral root	Sichuan
*Ginseng radix et rhizoma rubra*	Hongshen	*Panax ginseng C.A.Mey.*	Root and rhizome	Heilongjiang
*Zingiberis rhizoma*	Ganjiang	*Zingiber officinale Roscoe*	Rhizome	Sichuan
*Poria*	Fuling	*Poria cocos (Schw.)Wolf*	Dry sclerotia	Yunnan
*Schisandrae chinensis fructus*	Wuweizi	*Schisandra chinensis (Turcz.) Baill.*	Dry fruit	Liaoning
*Ophiopogonis radix*	Maidong	*Ophiopogon japonicus (Thunb.) Ker Gawl.*	Tuberous root	Sichuan
*Glycyrrhizae radix et rhizoma*	Gancao	*Glycyrrhiza uralensis Fisch. ex DC.*	Root and rhizome	Inner Mongolia

### 2.3 Extraction of metabolites from WZG

The WZG sample powder was weighed in a quantity of 50 mg using an analytical balance (BSA124S-CW, Sartorius, Germany). Following grinding, extraction, and homogenization, the WZG samples were placed in a refrigerator set to −40 °C for 30 min. The samples were then centrifuged at 12,000 rpm for 15 min at 4 °C, and the supernatant was extracted. Thereafter, the aforementioned steps were repeated. The supernatant was filtered using a microporous membrane with a pore size of 0.22 μm, and the filtered sample was employed in the subsequent assay.

### 2.4 UHPLC-OE-MS analysis

Ultra-high performance liquid chromatography-orbitrap exploris-mass spectrometry (UHPLC-OE-MS) analysis was conducted using a Vanquish UHPLC system from Thermo Fisher Scientific. The target compounds were chromatographically separated on a Phenomenex Kinetex C18 (2.1 mm × 100 mm, 2.6 μm) liquid chromatography column. The liquid chromatography phase A was an aqueous phase containing 0.01% acetic acid, while phase B was an isopropanol:acetonitrile (1:1, v/v) solution. The gradient elution conditions are detailed in the Table 2. The column temperature was set at 25 °C. The auto-sampler temperature was maintained at 4 °C, and the injection volume was 2 μL.

**TABLE 2:** Gradient elution process of UHPLC-OE-MS.

No	Time	Flow (mL/min)	%B
1	0.00	Run	
2	0.00	0.300	1.0
3	1.00	0.300	1.0
4	8.00	0.300	99.0
5	9.00	0.300	99.0
6	9.10	0.300	1.0
7	12.00	0.300	1.0
8	12.00	Stop Run	

### 2.5 Cell culture

The H9c2 cell line (AW-CNR083) was obtained from Changsha Abiowell Biotechnology Co., Ltd. (Changsha, China). H9c2 cells were cultured in Dulbecco’s Modified Eagle Medium (DMEM) (AW-MC006, Abiowell, China), supplemented with 10% fetal bovine serum (FBS) (10099141, Gibco, USA), 100 U/mL penicillin-streptomycin (AWH0529a, Abiowell, China), and 1.5 g/L NaHCO_3_. Once the cardiomyocytes had reached 80%–90% fusion, 0.25% trypsin was added, and the cells were passaged. The selected H9c2 cells, which were in the logarithmic growth phase, were placed in a 5% CO_2_ incubator at 37 °C for the subsequent experiments.

### 2.6 Preparation of drug-containing serum

WZG was initially prepared as a 6 mL solution in distilled water and subsequently administered to SPF-grade Sprague-Dawley (SD) male rats (aged 6–8 weeks, weighed 200–250 g) (Approval No.: SCXK (Xiang) 2019-0004) via gavage (1.44 g/kg/d) as previously described (Cai et al., 2019). The solution was administered via gavage at a dosage of 3 mL twice daily for a period of seven consecutive days. Two hours after the final dose, blood was collected from the abdominal aorta under anesthesia. The serum was obtained by centrifugation at 3,000 rpm for 10 min, heat-inactivated at 56 °C for 30 min to remove complement and bacteria, and then filtered through a 0.22 μm microporous membrane. The serum was aliquoted and stored at −20 °C. For *in vitro* studies, the drug-containing serum was added to the cell culture medium at a final concentration of 10% (v/v). A corresponding control group was treated with 10% blank serum from untreated rats.

### 2.7 Modeling of cardiomyocyte injury

DOX was utilized in the construction of an *in vitro* model for the examination of cardiomyocyte injury. A solution of 50 mM was prepared by dissolving 25 mg of DOX powder in dimethyl sulfoxide (DMSO) under sonication at 60 °C. The mother liquor was diluted 50-fold and formulated to a medium concentration of 1 mM. Subsequently, the diluted DOX master mix was combined with the cell culture medium, resulting in a final concentration of 2.67 μmol/L. The cardiomyocytes were cultured for 45 h in accordance with the methodology previously described (He et al., 2012).

### 2.8 Grouping and treatment

Cardiomyocytes were seeded in six-well plates, resulting in a total of four plates. Enalapril (ENP), a widely used ACE inhibitor in the clinical management of CHF, was used as a positive control to evaluate and compare the cardioprotective effect of WZG. The cells were separated into four distinct groups: the normal control group (NC), the DOX group (DOX), the DOX + drug-containing serum group (DOX+WZG), and the DOX + enalapril group (DOX+ENP).

### 2.9 Morphological observation

At 15, 30, and 45 h into the experiment, the morphological alterations of cardiomyocytes in each group were examined using an inverted biomicroscope (DSZ2000X, Beijing Zhongxian Hengye Instrument Co., Ltd., China).

### 2.10 CCK-8 assay

The viability of cardiomyocytes in each experimental group was determined using the CCK-8 method. The cardiomyocytes were initially collected, digested, and counted, and then inoculated in 48-well plates at a density of 1 × 10^4^ cells/well, with 300 μL per well. For each experimental group, three replicate wells were established. Subsequently, 30 μL of the CCK-8 solution was added to each well. The incubation was continued at 37 °C with 5% CO_2_ for a period of four hours. Afterwards, the optical density (OD) value at 450 nm was analyzed using a multifunctional enzyme marker (MB-530, Shenzhen Huisong Technology Development Co., Ltd., China).

### 2.11 Flow cytometry

The detection of apoptosis was conducted using flow cytometry in each group. The cardiomyocytes were initially washed twice with phosphate-buffered saline (PBS) and subsequently subjected to centrifugation at 2000 rpm for a period of 5 minutes on each occasion, after which they were collected. To achieve an adequate suspension of the cells, a volume of 500 µL of binding buffer was added. Subsequently, 5 µL of Annexin V-APC and 5 µL of propidium iodide solution were added and thoroughly mixed. The reaction was conducted at room temperature in the absence of light for a period of 10 min. Afterwards, the apoptosis rate was determined by flow cytometry (A00-1-1102, Beckman, USA). The flow cytometry data were analyzed using CytExpert software (Beckman Coulter). Cell debris was excluded based on forward scatter (FSC) and side scatter (SSC) profiles. Single cells were selected using FSC-A vs. FSC-H gating. Apoptotic cells were identified based on Annexin V-APC and PI staining patterns: Annexin V^+^/PI^−^ cells were classified as early apoptotic, Annexin V^+^/PI^+^as late apoptotic or necrotic, Annexin V^−^/PI^+^ as necrotic, and Annexin V^−^/PI^−^ as viable. Compensation was performed using single-stained controls.

### 2.12 Extraction of exosomes

The cell supernatant was collected and subjected to centrifugation at 1500 rpm for a period of 5 minutes. Thereafter, the floating cells were removed. Subsequently, the supernatant was subjected to centrifugation at 3,000 rpm for 15 min, after which the cell debris was removed. Afterwards, the supernatant was filtered through a 0.22 um filter membrane. The cell supernatant was subjected to ultrafiltration using an ultrafiltration tube (UFC910096-1pk, Millipore, USA) and subsequently centrifuged at 3,000 rpm for 10 min. Finally, the supernatant of the enriched cells was harvested and subjected to centrifugation at 100,000 × g for a period of 2 hours. The precipitate obtained following the removal of the supernatant was identified as exosomes.

### 2.13 Transmission electron microscopy (TEM)

A volume of 15 μL of exosome suspension was transferred to a 200-mesh copper grid, and the filtrate was allowed to stand for a period of 2 minutes before being blotted dry. A solution of uranyl acetate at a concentration of 2% was applied for a period of 15 s. Subsequently, the copper grid was permitted to dry naturally at room temperature and was then mounted on a transmission electron microscope (JEM-2100, JEOL, Japan) operating at 100 kV. The morphological characteristics of exosomes were observed under a TEM.

### 2.14 Nanoparticle tracking analysis (NTA)

The analysis of the particle size distribution and concentration of isolated EVs was conducted using a NanoSight NS300 nanoparticle tracking analyzer (Malvern Instruments, UK) with software version NTA 3.4 Build 3.4.4. Prior to analysis, exosome samples were moderately diluted with particle-free PBS to approximately 20–50 particles per frame to avoid statistical bias caused by excessive particle aggregation or dilution. During the analysis, a red laser was utilized as the light source. The camera level was set to 14, the detection threshold to 5, and the frame rate to 25 frames per second. Three to five random video segments (60–90 s each) were captured per sample, with temperature automatically recorded by the instrument (approximately 24 °C–25 °C). The analysis of all videos is conducted using identical parameters to obtain the mean particle size (Mean), median size (D50), mode particle size (Mode), standard deviation (SD), and particle concentration (particles/mL).

### 2.15 Western blot

Western blot was employed to ascertain the expression levels of CD9 and CD63 in exosomes, as well as the expression levels of p38 MAPK, p-p38 MAPK, Caspase-3, Bcl-2, and Bax in cardiomyocytes. Total cellular proteins were extracted with 200 µL of RIPA lysis buffer, and the protein concentration was subsequently determined in accordance with the instructions provided with the BCA protein assay kit. The requisite quantity of protein samples was separated by SDS-PAGE electrophoresis, and the membrane was transferred at a constant voltage of 80 V and incubated in closed mode for 90 min. The primary antibodies were diluted at a ratio of 1: 1000, and the *β*-actin antibody was diluted at a ratio of 1: 5000. The membrane was incubated with the primary antibody at 4 °C overnight. The horseradish peroxidase-labeled secondary antibodies were diluted at a ratio of 1: 5000, and the membrane was incubated with the secondary antibodies for 90 min at room temperature. The electrochemiluminescence (ECL) reagent was utilized for color development and exposure. Subsequently, the gray scale values of the protein bands were analyzed using the ImageJ software (version 1.53).

### 2.16 Real time-quantitative polymerase chain reaction (RT-qPCR)

Total RNA was extracted using the Trizol method, and the RNA concentration was determined by UV spectrophotometry. The cDNA was synthesized in adherence to the instructions prescribed by the HiFiScript cDNA Synthesis kit, and the resulting cDNA was employed as a template for amplification using the UltraSYBR Mixture Synthesis kit, as per the instructions provided. The gene sequences of miR-155, p38 MAPK, Bcl-2, and Bax were obtained from the National Center for Biotechnology Information (NCBI) database. Primers were designed using the Primer5 software and subsequently synthesized by Beijing Tsingke Biotech Co., Ltd (Table 3). The analysis was conducted in accordance with the instructions provided in the PCR reaction kit. The expression levels of miR-155 in exosomes and the mRNA levels of miR-155, p38 MAPK, Bcl-2, and Bax in cardiomyocytes were determined via the 2^−ΔΔCt^ method. 5S was utilized as the internal reference for miR-155, whereas the remaining mRNA internal reference was GAPDH. Quantitative PCR amplification was conducted under the following conditions: an initial denaturation at 95 °C for 10 min, followed by 40 cycles of denaturation at 95 °C for 15 s and annealing at 60 °C for 30 s.

**TABLE 3:** Primer design for RT-qPCR.

Gene	Forward primer	Reverse primer
miR-155	5′-TTA​ATG​CTA​ATT​GTG​ATA​GGG​GT-3′	3′-GCT​GTC​AAC​GAT​ACG​CTA​CGT​A-5′
p38 MAPK	5′-AGC​TTA​CCG​ATG​ACC​ACG​TT-3′	3′-CAC​GTA​GCC​GGT​CAT​TTC​GTC-5′
Bcl-2	5′-CTG​GTG​GAC​AAC​ATC​GCT​CT-3′	3′-ATA​GTT​CCA​CAA​AGG​CAT​CCC​A-5′
Bax	5′-TTG​CTA​CAG​GGT​TTC​ATC​CAG​G-3′	3′-GCT​CCA​AGG​TCA​GCT​CAG​GT-5′
5S	5′-GCC​TAC​AGC​CAT​ACC​ACC​CGG​AA-3′	3′-CCT​ACA​GCA​CCC​GGT​ATC​CCA-5′
GAPDH	5′-ACA​GCA​ACA​GGG​TGG​TGG​AC-3′	3′-TTT​GAG​GGT​GCA​GCG​AAC​TT-5′

### 2.17 Statistical analysis

The data were analyzed using GraphPad Prism (version 9.4.1) software, and the results are expressed as mean ± standard deviation (SD). A one-way analysis of variance (ANOVA) was employed to ascertain whether a significant discrepancy existed between the groups. A *p*-value of less than 0.05 indicated the presence of statistically significant differences.

## 3 Results

### 3.1 Chemical composition of WZG

**FIGURE 1:**
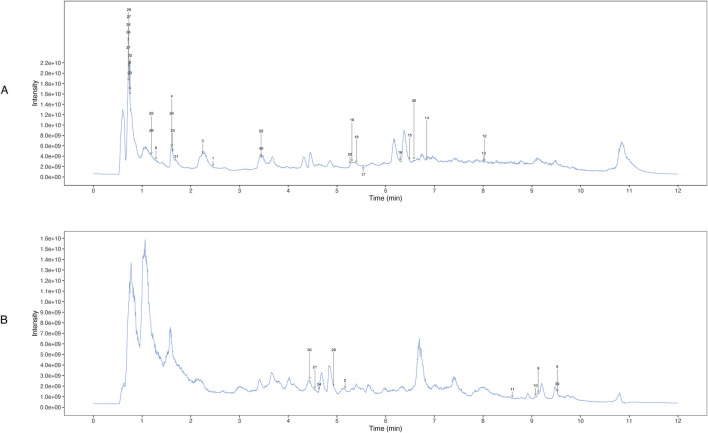
Determination of chemical constituents of WZG by UHPLC-OE-MS. **(A)** TIC peak of the sample in positive ion mode; **(B)** TIC peaks of samples in negative ion mode.

UHPLC-OE-MS analysis revealed that WZG encompass 1,147 distinct chemical components, which could be classified into 178 distinct categories. Among the 1,147 components identified via UHPLC-OE-MS, ginsenosides Rg1, Rb1, and Re were selected as the primary bioactive components of WZG based on their relative abundance, reported pharmacological activity in the literature, and network pharmacology analysis. These compounds have been reported to play important roles in cardiovascular protection by regulating oxidative stress, mitochondrial function, apoptosis, and inflammation-related pathways such as the PI3K/AKT, p53, and MAPK pathways (Yao et al., 2021). The twenty most abundant substances were selected for TIC icon peak examples based on their scoring values. Figure 1 illustrates the UHPLC-OE-MS profile of WZG in negative/positive ion mode. The 40 peaks identified in the plot were predominantly composed of compounds belonging to the categories of cyclic polyketides, flavonoids, saccharides, fatty acids and conjugates, isoflavonoids, lignans, terpenoids, and small peptides (Table 4).

**TABLE 4:** Metabolites of Wenyang Zhenshuai Granules by UHPLC-OE-MS.

ID	Identification	Formula	Measured (m/z)	Ion mode (m/z)	Retention time (s)	ppm
12	Schisandrin B	C23H28O6	401.1957	[M+H]+	481.5	0.4
13	[8]-Shogaol	C19H28O3	305.211	[M+H]+	480.7	0.6
14	Semilicoisoflavone B	C20H16O6	353.1018	[M+H]+	410.5	0.6
35	(24S,25R)-12,25-dihydroxy-18,19,20-trimethoxy-11,12,24,25-tetramethyl-4,6,9,14-tetraoxapentacyclo[13.7.3.03,7.08,22.016,21]pentacosa-1,3(7),8(22),16,18,20-hexaen-13-one	C28H34O10	531.2219	[M+H]+	394.8	1.1
15	Medicarpin	C16H14O4	271.0962	[M+H]+	389.4	1.1
16	Nobiletin	C21H22O8	403.1384	[M+H]+	377.8	1.0
17	Hexahydrocurcumin	C21H26O6	375.1801	[M+H]+	332.3	0.4
18	N-trans-Feruloyltyramine	C18H19NO4	314.1385	[M+H]+	323.9	0.7
19	Ononin	C22H22O9	431.1333	[M+H]+	318.5	0.9
20	Isoliquiritin apioside	C26H30O13	551.1755	[M+H]+	316	0.8
22	Tryptophan	C11H12N2O2	205.0973	[M+H]+	206.5	0.5
40	DL-Tryptophan	C11H12N2O2	205.0973	[M+H]+	206.5	0.5
1	2-methyl-3-[(2S,3R,4S,5S,6R)-3,4,5-trihydroxy-6-(hydroxymethyl)tetrahydropyran-2-yl]oxy-pyran-4-one	C12H16O8	289.0918	[M+H]+	147.4	0.0
3	3-methyl-2,3,6,7,8,8a-hexahydropyrrolo[1,2-a]pyrazine-1,4-dione	C8H12N2O2	169.0971	[M+H]+	134.8	0.7
31	5-Hydroxymethylfurfural	C6H6O3	127.039	[M+H]+	102	0.3
23	Kojic acid	C6H6O4	143.0339	[M+H]+	97.9	0.3
4	Matrine	C15H24N2O	249.1962	[M+H]+	96.5	0.3
24	Anabasine	C10H14N2	163.123	[M+H]+	96.5	0.1
6	Adenine	C5H5N5	136.0618	[M+H]+	77.1	0.2
25	Leucine	C6H13NO2	132.1019	[M+H]+	71.5	0.3
26	Norleucine	C6H13NO2	132.1019	[M+H]+	71.5	0.3
32	Proline	C5H9NO2	116.0705	[M+H]+	45.2	1.0
33	D-Proline	C5H9NO2	116.0705	[M+H]+	45.2	1.0
5	Trigonelline	C7H7NO2	138.0549	[M+H]+	44.9	0.7
27	Trehalose	C12H22O11	365.1053	[M+Na]+	43.8	0.6
28	Melibiose	C12H22O11	365.1053	[M+Na]+	43.8	0.6
7	Galactose	C6H12O6	203.0525	[M+Na]+	43.1	0.9
36	Tagatose	C6H12O6	203.0525	[M+Na]+	43.1	0.9
37	Fructose	C6H12O6	203.0525	[M+Na]+	43.1	0.9
38	Allose	C6H12O6	203.0525	[M+Na]+	43.1	0.9
8	Oleic acid	C18H34O2	281.2483	[M-H]-	571.3	1.1
39	trans-Vaccenic acid	C18H34O2	281.2483	[M-H]-	571.3	1.1
9	cis-9-Palmitoleic acid	C16H30O2	253.2171	[M-H]-	547.9	0.9
10	Myristic acid	C14H28O2	227.2014	[M-H]-	544.4	0.9
11	Stearidonic acid	C18H28O2	275.2014	[M-H]-	516	0.8
2	Choerospondin	C21H22O10	433.1139	[M-H]-	310	0.1
29	Suberic acid	C8H14O4	173.0818	[M-H]-	295.9	0.7
34	Vanillin	C8H8O3	151.04	[M-H]-	277.8	0.5
21	Vicenin-1	C26H28O14	563.1406	[M-H]-	272.9	0.0
30	Caffeic acid	C9H8O4	179.0348	[M-H]-	266.2	0.6

### 3.2 WZG improves the morphology of cardiomyocytes in CHF

**FIGURE 2:**
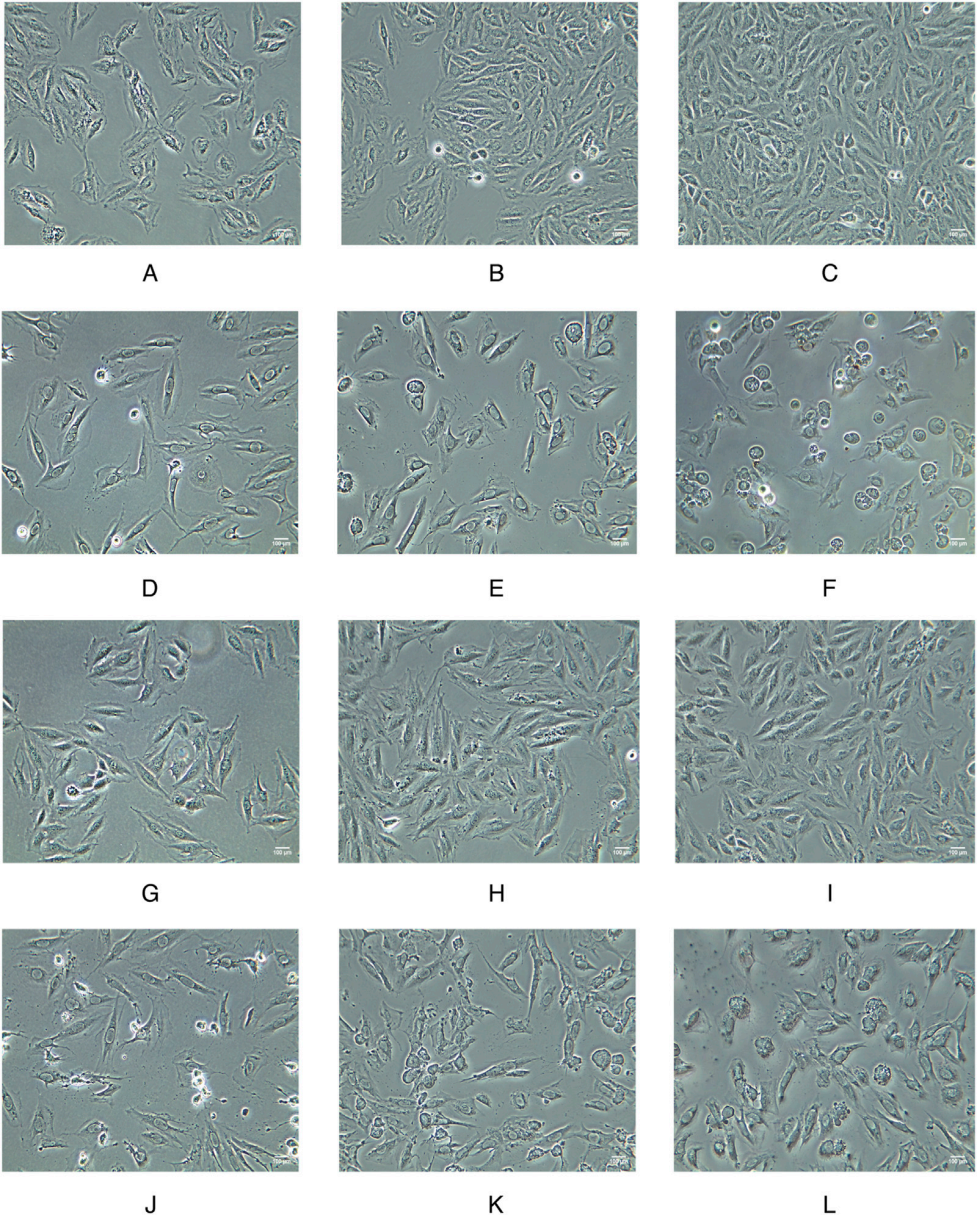
Morphological images of cardiomyocytes from each group at 15, 30, and 45 h of the experiment (×100). **(A–C)** the normal control group; **(D–F)** the doxorubicin group; **(G–I)** the doxorubicin + drug-containing serum group; **(J–L)** the doxorubicin + enalapril group.

To investigate the impact of WZG on the structural characteristics of cardiomyocytes in CHF, we conducted a detailed examination of the cellular morphology in each experimental group at 15, 30, and 45 h into the study period (Figure 2). The cells in the NC group exhibited optimal growth status, with clear borders, regular arrangement, and a long shuttle-shaped morphology. Moreover, the cell density demonstrated a significant increase with the prolongation of the incubation period. In contrast to the NC group, the DOX group exhibited a notable decline in cellular growth status. The cell borders were indistinct, the arrangement was disorganized, and evidence of myofiber dissolution and cytoplasmic vacuolar degeneration was apparent. As the duration of the intervention involving the WZG-containing drug serum was prolonged, the cell morphology of the DOX + WZG group exhibited a progressive return to a normal state. The edges of the cells became increasingly clear, the cytosol gradually filled, the cell arrangement became increasingly regular in comparison to the initial state. The aforementioned results demonstrate that WZG is capable of reversing the morphological alterations of cardiomyocytes in a CHF model induced by DOX.

### 3.3 WZG increases cardiomyocyte viability and decrease apoptosis rate

**FIGURE 3:**
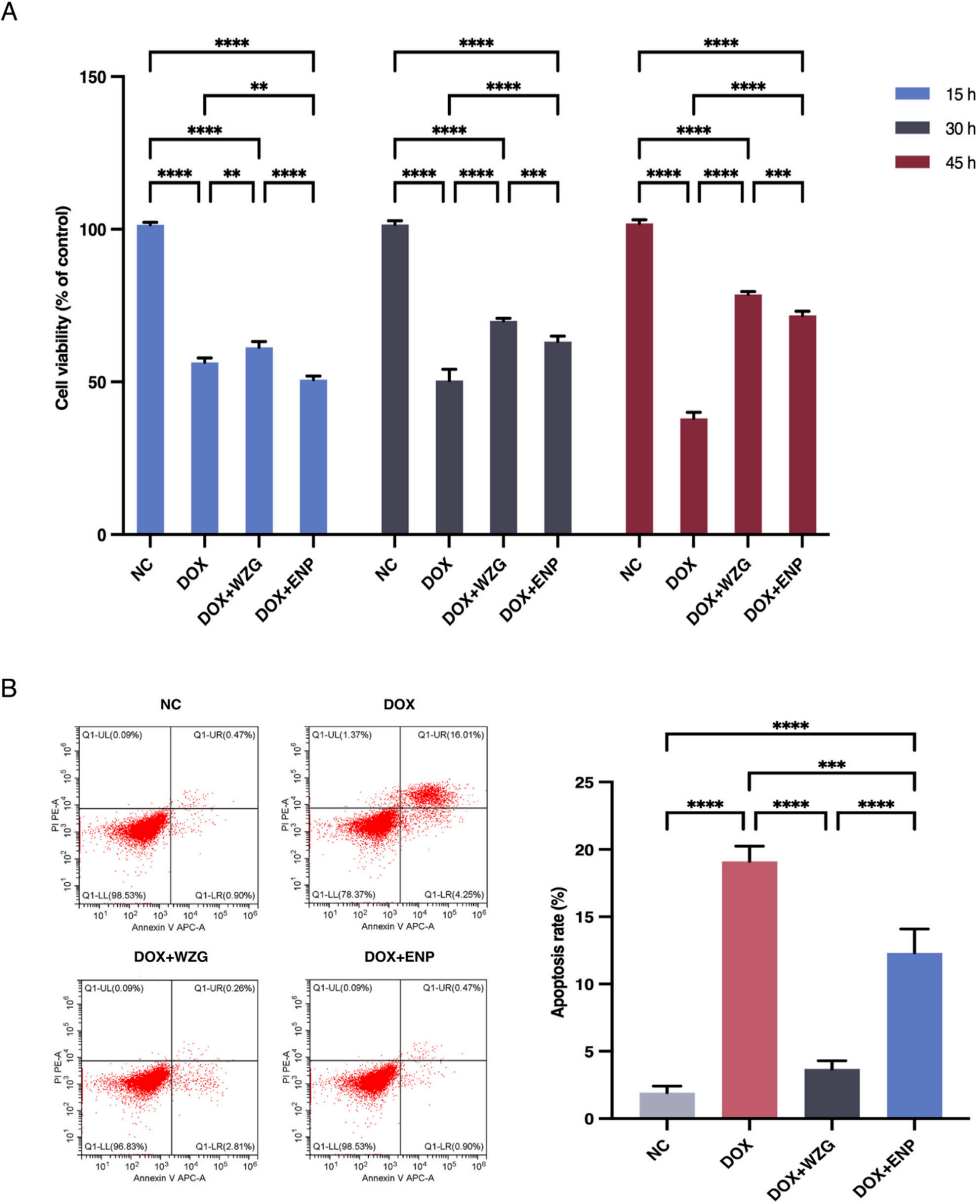
Determination of cardiomyocyte viability and apoptosis rate. **(A)** Cell viability of cardiomyocytes in each group at 15, 30, and 45 h of the experiment; **(B)** Apoptosis rate of cardiomyocytes in each group detected by flow cytometry. NC: the normal control group; DOX: the doxorubicin group; DOX + WZG: the doxorubicin + drug-containing serum group; DOX + ENP: the doxorubicin + enalapril group. **P < 0.01, ***P < 0.001, ****P < 0.0001.

To determine the impact of WZG on cardiomyocyte viability in a model of DOX-induced CHF, we utilized the CCK-8 assay to assess the proliferative activity of cells at 15, 30, and 45 h post-treatment (Figure 3A). In comparison to the NC group, the DOX group exhibited a significant decline in cell viability. In contrast to the DOX group, the cardiomyocyte viability in the DOX + WZG group was markedly elevated, exhibiting a positive correlation with the intervention time. To further substantiate the impact of WZG on cell apoptosis, the number of apoptotic cells was quantified through flow cytometry at the 45th hour of the experiment (Figure 3B). Following the administration of WZG-containing serum, a notable reduction in the apoptosis rate was observed in the DOX + WZG group. These findings indicate that WZG can markedly enhance the viability of H9c2 cells and potently suppressed DOX-induced cell apoptosis.

### 3.4 WZG promotes exosomal microRNA-155 secretion from cardiomyocytes

**FIGURE 4:**
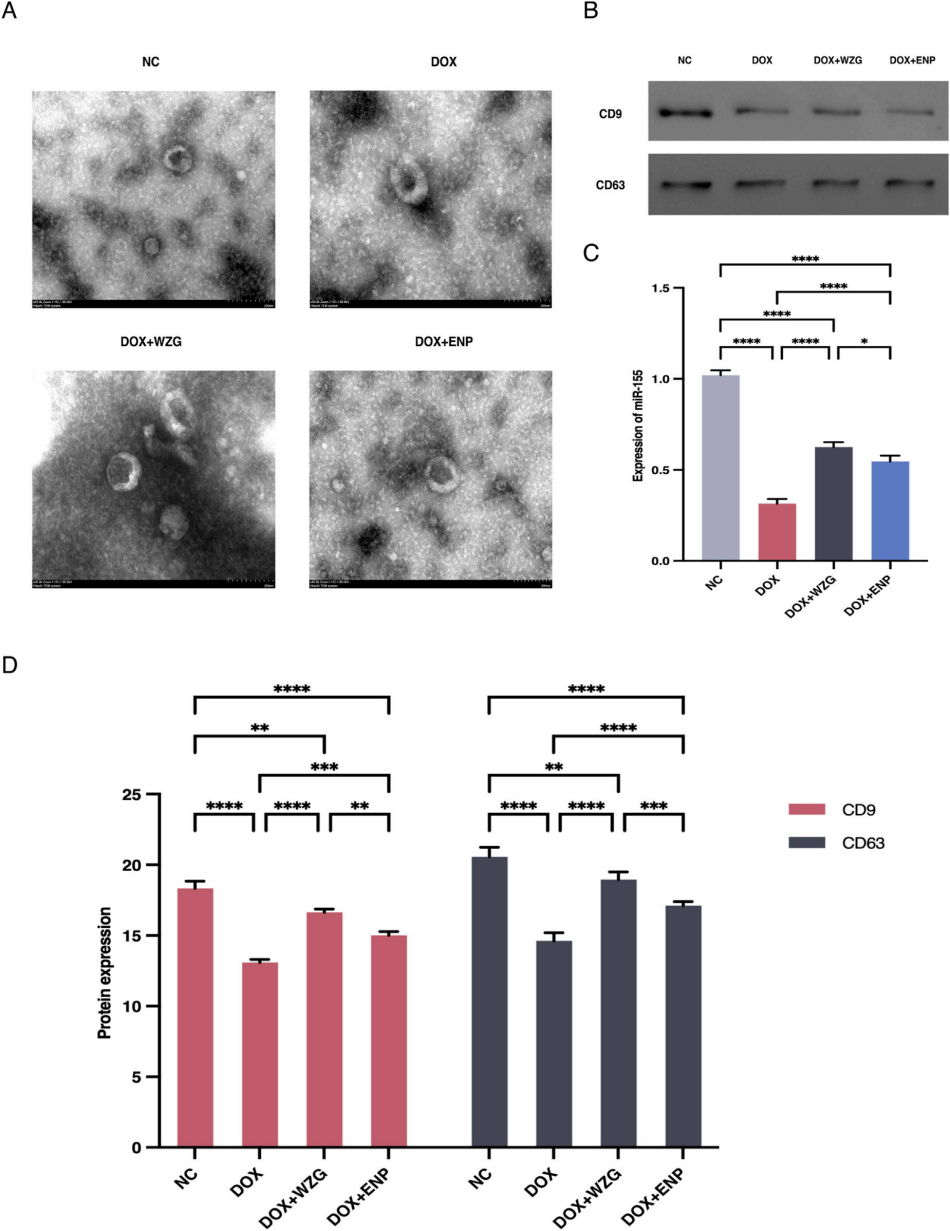
Identification of exosomes and expression levels of miR-155 in exosomes. **(A)** Morphological characterization of exosomes (×50); **(B)** Protein strip charts for CD9 and CD63; **(C)** Expression levels of miR-155 in exosomes; **(D)** Expression of exosome marker proteins CD9 and CD63 detected by Western blot. NC: the normal control group; DOX: the doxorubicin group; DOX + WZG: the doxorubicin + drug-containing serum group; DOX + ENP: the doxorubicin + enalapril group. *P < 0.05, **P < 0.01, ***P < 0.001, ****P < 0.0001.

**FIGURE 5:**
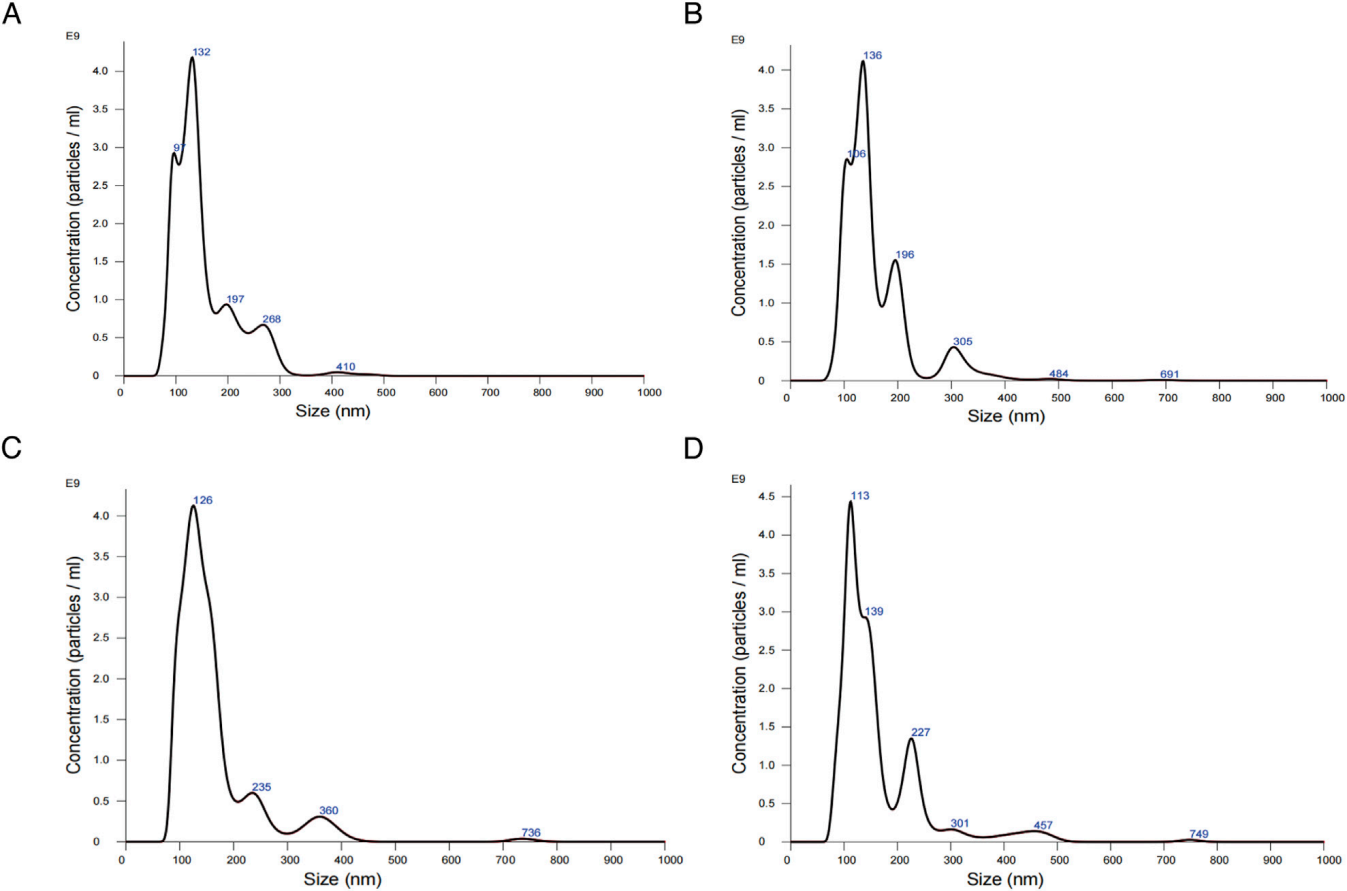
Results of nanoparticle tracking analysis for exosomes. **(A)** the normal control group; **(B)** the DOX group; **(C)** the DOX + drug-containing serum group; **(D)** the DOX + enalapril group.

To examine the influence of WZG on the secretion of exosomal miR-155 from cardiomyocytes, we initially extracted exosomes derived from H9c2 cells from serum cultures and observed the morphology and size of the exosomes under a TEM (Figure 4A). Examination under the microscope revealed that the morphological features of exosomes exhibited a typical round or oval vesicular structure with an intact double-layered envelope structure containing low-electron-density granular material. NTA revealed that exosomes in the DOX + WZG group exhibited an average particle size of 161.8 nm, a D50 of 139.0 nm, and a particle concentration of 3.69 × 10^11^ particles/mL, which was higher than that in the DOX model group (average particle size 153.6 nm, D50 134.7 nm, concentration 3.39 × 10^11^ particles/mL). Compared to the normal group (mean particle size 159.1 nm, D50 138.6 nm, concentration 3.14 × 10^11^ particles/mL) and the enalapril group (mean particle size 164.7 nm, D50 136.9 nm, concentration 3.45 × 10^11^ particles/mL), the DOX + WZG group exhibited significantly increased exosome concentration while maintaining particle size within the normal exosome range (Figure 5). These results suggest that WZG treatment promotes the secretion of exosomes from cardiomyocytes. Subsequently, the expression of exosome marker proteins, namely CD9 and CD63, was analyzed via Western blot. The results demonstrated that the protein expression levels of CD9 and CD63 were markedly elevated in the DOX + WZG group relative to the DOX group (Figures 4B,D). Additionally, the RT-qPCR method was utilized to further ascertain the content of miR-155 in exosomes (Figure 4C). The results revealed a notable elevation in the expression of miR-155 in exosomes within the DOX + WZG group. The aforementioned results indicate that WZG may facilitate the secretion of exosomes from cardiomyocytes and enhance the concentration of miR-155 within these exosomes.

### 3.5 WZG inhibits cardiomyocyte apoptosis by regulating p38 MAPK protein phosphorylation via exosomal miR-155

**FIGURE 6:**
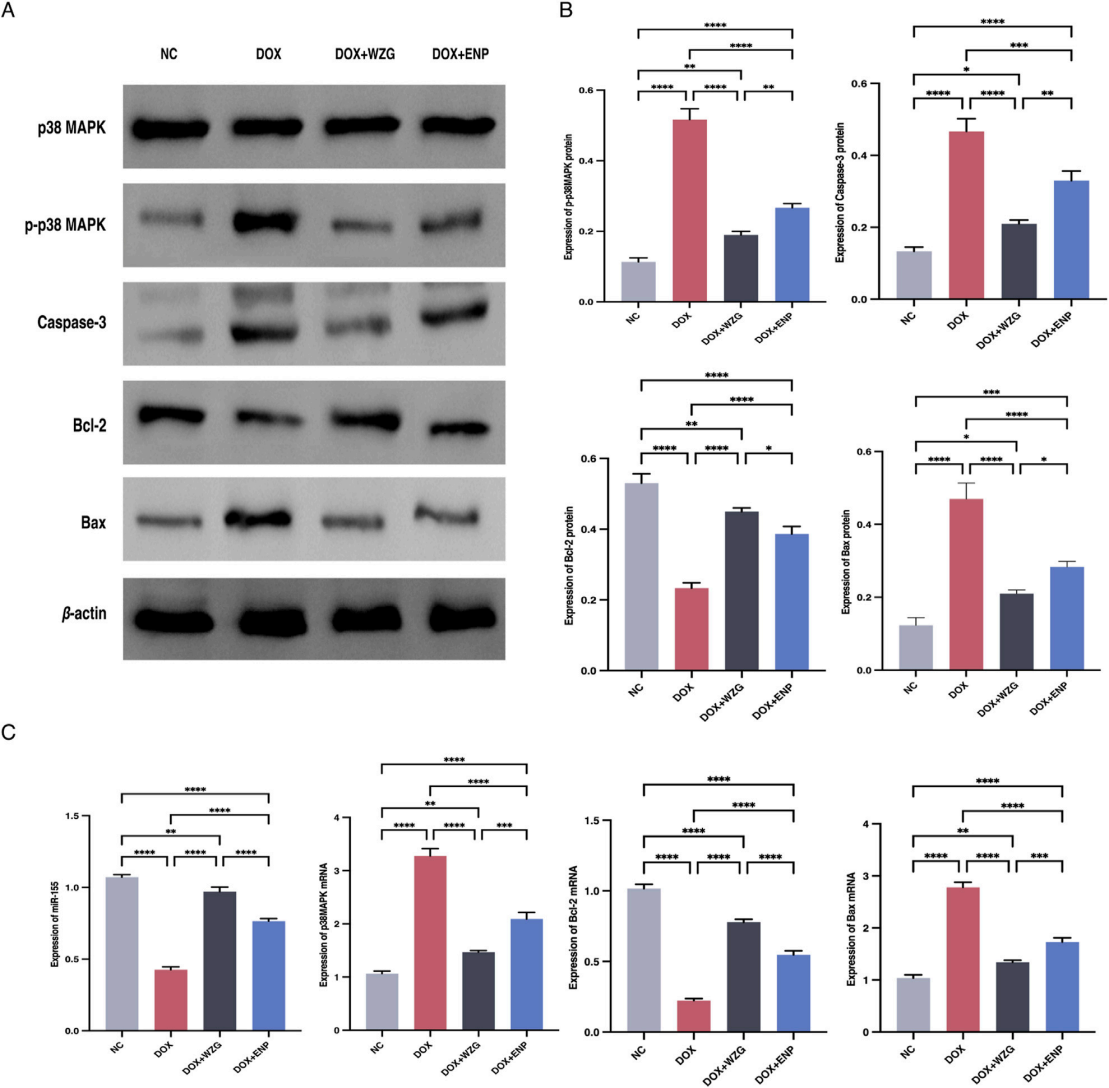
Results of Western blot and PT-qPCR assays. **(A)** Protein strip charts for p38 MAPK, p-p38 MAPK, Caspase-3, Bcl-2, and Bax; **(B)** Expressions ofp38 MAPK, p-p38 MAPK, Caspase-3, Bcl-2 and Bax detected by Western blot; **(C)** Expression of miR-155, p38 MAPK mRNA, Bcl-2 mRNA and Bax mRNA detected by RT-qPCR. NC: the normal control group; DOX: the doxorubicin group; DOX + WZG: the doxorubicin + drug-containing serum group; DOX +ENP: the doxorubicin + enalapril group. *P < 0.05, **P < 0.01, ***P < 0.001, ****P < 0.0001.

To clarify the underlying regulatory mechanism of WZG on the p38 MAPK pathway and cell apoptosis, Western blot was employed to assess the expression of p38 MAPK, p-p38 MAPK, Caspase-3, Bcl-2, and Bax in cardiomyocytes. Additionally, the expression of miR-155, p38 MAPK mRNA, Bcl-2 mRNA, and Bax mRNA in cells were investigated through RT-qPCR (Figures 6A,B). In the DOX + WZG group, there was a notable reduction in the expression levels of p-p38 MAPK, Caspase-3, and Bax proteins, while the expression level of Bcl-2 exhibited a significant increase. The RT-qPCR results were in accordance with those obtained from the Western blot analysis (Figure 6C). The administration of WZG was observed to inhibit p38 MAPK protein phosphorylation, thereby modulating the expression of apoptotic proteins. Moreover, the elevated expression of miR-155 was demonstrated to promote cardiomyocyte survival and was found to be closely associated with p38 MAPK protein phosphorylation. It is hypothesized that WZG may inhibit p38 MAPK phosphorylation by upregulating the expression of exosomal miR-155, thereby exerting an anti-apoptotic effect on cardiomyocytes.

## 4 Discussion

CHF represents the terminal stage of progression in the development of a range of CVDs. Despite the continuous updating of modern medical diagnosis and treatment strategies, the mortality and rehospitalization rates of patients with CHF remain high. In recent years, TCM has been demonstrated to possess notable clinical characteristics and therapeutic advantages in the treatment of CHF. WZG is a TCM compound formula that has been demonstrated to be efficacious in the treatment of CHF. Recent studies suggest that these benefits may be linked to its regulation of apoptosis and related molecular pathways. The present study demonstrates that WZG can inhibit apoptosis in CHF by promoting the secretion of exosomes from cardiomyocytes, increasing the content of exosomal miR-155, and inhibiting the p38 MAPK signaling pathway.

Exosomes are instrumental in the intercellular transfer of signaling molecules, including nucleic acids, proteins, and lipids, which are essential for intercellular interactions (Gurung et al., 2021). MicroRNAs constitute a principal component of exosomes (Cunha et al., 2024). Upon release into the extracellular space, exosomes can transfer miRNAs to neighboring or distant cells, thereby exerting significant pathophysiological effects and participating in a multitude of biological processes (Du et al., 2021). At present, exosomal miRNA-mediated intercellular communication plays a significant role in the pathogenesis of CHF, particularly in the inhibition of cardiomyocyte hypertrophy, attenuation of myocardial fibrosis (MF), and regulation of myocardial angiogenesis (Li K. et al., 2023; Xue et al., 2020; Zheng et al., 2020). Moreover, peripheral circulating exosomes, exosomes derived from mesenchymal stem cells, and exosomes derived from cardiomyocytes represent a promising avenue for clinical therapeutic strategies in the treatment of CHF (Xiao et al., 2022; Yan et al., 2022; Yuan et al., 2023).

The differential expression of miR-155 has been evidenced to be related to the pathogenesis of CVDs. miR-155 affects pathological processes such as the inflammatory response, fibrosis, and apoptosis in the heart by binding to target mRNAs and regulating target genes related to cardiovascular function. Our previous study demonstrated that the overexpression of miR-155 inhibited cell apoptosis and ameliorated cardiac functional impairment in mice with CHF by regulating hypoxia-inducible factor 1 alpha (HIF-1α) (Luo et al., 2023). Additionally, miR-155 has been recognized as a principal mediator of myocardial inflammation, with its expression markedly elevated in inflammatory responses associated with CHF (Oh et al., 2022; Ge et al., 2021). Moreover, miR-155 has been observed to exert regulatory effects on MF. The knockdown of the miR-155 gene has been demonstrated to inhibit the proliferation of cardiac fibroblasts, reduce infarct size, and decrease collagen deposition, thereby attenuating VR in CHF (He et al., 2016). Meanwhile, the downregulation of miR-155 expression has been proven to exert a protective effect against apoptosis in H9c2 cells. Inhibition of miR-155 expression has been shown to attenuate cardiac dysfunction and apoptosis induced by lipopolysaccharide (LPS) or ischemia/reperfusion (I/R) in previous studies (Wang et al., 2016; Hu et al., 2023).

The p38 MAPK pathway plays a critical role in cellular stress responses and inflammation and is closely associated with cardiomyocyte apoptosis. Moreover, it is implicated in a multitude of physiopathological processes, including cardiac and vascular smooth muscle cell proliferation, endothelial cell injury, myocardial hypertrophy, and extracellular matrix synthesis and degradation (Martínez-Limón et al., 2020; Marber et al., 2011; Martin et al., 2015). Additionally, p38 MAPK functions as a stress signaling pathway that regulates apoptosis in cardiomyocytes. Selective inhibition of p38 MAPK has been demonstrated to ameliorate cell apoptosis associated with CHF, myocardial I/R, and myocardial infarction (MI) (Li et al., 2006; Ma et al., 1999; Wang et al., 1998). The role of the p38 MAPK signaling pathway and miRNAs in the context of CVDs has recently emerged as a topic of considerable interest among researchers both domestically and internationally. It has been evidenced that the upregulation of miR-19 enhances cardiac function and mitigates MF by suppressing the MAPK signaling pathway (Song et al., 2023). Similarly, miR-1283 and miR-20a have been elucidated to mitigate hypoxia/reoxygenation-induced cardiomyocyte injury by inhibiting p38 MAPK (Liu et al., 2021; Gong and Zhang, 2019). These findings support our hypothesis that exosomal miR-155 may exert cardioprotective effects by suppressing p38 MAPK activation.

In this study, WZG was found to promote exosome secretion and increase the loading of miR-155 into exosomes. The precise mechanisms underlying exosome biogenesis and secretion remain to be elucidated. However, recent studies have identified the involvement of Rab GTPases, neutral sphingomyelinase 2 (nSMase2), and ESCRT machinery in the regulation of these processes (Colombo et al., 2014; Trajkovic et al., 2008). The potential influence of WZG on these processes warrants further investigation. Moreover, studies have demonstrated that the selective loading of microRNAs into exosome particles is contingent on the presence of RNA-binding proteins, including hnRNPA2B1, SYNCRIP, and AGO2. These proteins have been demonstrated to recognize specific sequence motifs present on the miRNAs, thereby facilitating their incorporation into exosome particles (Villarroya-Beltri et al., 2013; Santangelo et al., 2016; Guduric-Fuchs et al., 2012). Therefore, it is plausible that WZG enhances miR-155 content by modulating these RNA-sorting proteins or by increasing miR-155. Further studies are warranted to investigate these potential mechanisms.

In this study, we employed DOX to establish an *in vitro* model of cardiomyocyte injury. DOX-induced cardiomyocyte injury is a widely used model for studying the mechanisms of cardiac dysfunction. This model primarily reflects a drug-induced form of cardiomyopathy rather than the full range of causes of CHF, such as ischemic, hypertensive, or valvular heart disease. In the context of our research, the emphasis on the miR-155/p38 MAPK signaling axis and its role in apoptosis establishes a controllable and reproducible platform for the dissection of these molecular mechanisms using the DOX model. However, this model may not fully recapitulate all the etiological and hemodynamic features of human CHF. Consequently, subsequent studies using pressure overload, ischemia-induced, or volume overload CHF models will be necessary to validate our findings.

Among the 1,147 compounds identified by UHPLC-OE-MS, ginsenosides Rg1, Rb1, and Re are the components of particular interest. Previous studies have shown that ginsenoside Rg1 can prevent and treat CHF by downregulating ERK1/2 protein phosphorylation or inhibiting heme synthesis to increase succinyl CoA, thereby promoting mitochondrial homeostasis (Peng et al., 2024; Chen et al., 2025). Ginsenoside Rb1 has been observed to exert a positive effect on HF by modulating the FADD and PPARα pathways (Li C. et al., 2023). Furthermore, ginsenoside Re has been documented to mitigate myocardial injury by regulating the miR-489/MyD88/NF-κB axis (Sun et al., 2023). In addition to ginsenosides, several bioactive compounds identified by UHPLC-OE-MS analysis may also contribute to the therapeutic effects of WZG in CHF. As stated in the extant literature, Schisandrin B has been shown to alleviate angiotensin II-induced cardiac inflammatory remodeling by inhibiting the recruitment of MyD88 to TLR in mouse cardiomyocytes (Xu et al., 2024). Nobiletin has demonstrated potential for treating HF by preventing the development of doxorubicin-induced HF through the inhibition of apoptosis (Sunagawa et al., 2017; Sunagawa et al., 2025). A multitude of studies have demonstrated that [8]-Shogaol exerts effective anti-inflammatory and anti-apoptotic effects by inhibiting NF-κB and MAPK signaling pathways (Jo et al., 2022). Collectively, these findings suggest that WZG’s therapeutic effects in CHF may stem from the synergistic actions of multiple bioactive components.

Compared with enalapril, WZG demonstrated a more pronounced cardioprotective effect by not only improving cell viability and reducing apoptosis but also more effectively modulating key signaling pathways, such as p38 MAPK phosphorylation and the expression of apoptotic proteins. Although enalapril demonstrated a modest increase in exosomal miR-155, the effect was considerably more pronounced in the WZG group. These results suggest that the enhanced efficacy of WZG may be partially attributed to its augmented capacity to promote exosome-mediated delivery of miR-155, thereby highlighting a mechanistic advantage that complements conventional RAAS inhibition.

In this study, we employed DOX to develop an *in vitro* model of cardiomyocyte injury with the objective of elucidating the potential mechanism of action of WZG in inhibiting cell apoptosis through the exosomal miR-155/p38MAPK signaling pathway. The results demonstrated that WZG significantly improved cardiomyocyte morphology, enhanced cell viability, and reduced apoptosis, consistent with the findings of the research group’s previous studies. Mechanistically, WZG promoted the secretion of exosome from cardiomyocytes and markedly increased the expression of microRNA-155 within these exosome. This microRNA, which has been demonstrated to regulate apoptosis-related genes, can be internalized by neighboring cells via exosomal transport (You et al., 2025). Elevated levels of exosomal miR-155 have been demonstrated to inhibit the phosphorylation of p38 MAPK, downregulate pro-apoptotic proteins such as Bax, and upregulate anti-apoptotic proteins like Bcl-2 (Ou et al., 2019). Therefore, our findings suggest that WZG exerts anti-apoptotic effects on cardiomyocytes in part by increasing exosomal miR-155 expression and suppressing the p38 MAPK signaling pathway.

Despite the promising results, this study has several limitations. First, the relatively high dose of WZG (1.44 g/kg/day) was derived from traditional body surface area (BSA) conversion, a method increasingly scrutinized for potentially producing supra-therapeutic exposures, especially for multi-component herbal preparations (Reagan-Shaw et al., 2008; Heinrich et al., 2020). Although significant biological effects were observed, these may partially result from non-specific activity. Future studies should aim to define the minimal effective dose and conduct pharmacokinetic profiling to determine a clinically meaningful dosage range. Secondly, the study employed a single dose level, which restricts the interpretation of dose-response relationships. The initial design was conceived with the objective of assessing biological activity. However, subsequent studies should encompass multiple dose levels and pharmacokinetic parameters. Thirdly, although UHPLC-OE-MS and validated HPLC methods were employed to characterize major ginsenosides (Yang et al., 2017), current guidelines recommend at least three orthogonal analytical approaches. Consequently, expanded phytochemical profiling is planned in accordance with the ConPhyMP framework. In addition, *in vivo* animal experiments will be conducted to further verify the cardioprotective effect of WZG and to elucidate the role of exosomal miR-155 in the context of CHF. Finally, while WZG promoted the release of miR-155-enriched exosome that may be internalized by cardiomyocytes to inhibit p38 MAPK phosphorylation and apoptosis, direct evidence for exosome uptake and intracellular activity remains lacking. Subsequent studies that utilize exosome-tracking and miR-155-targeted interventions are required to validate this mechanism.

## 5 Conclusion

In brief, the findings of this study demonstrate that WZG exerts an interventional effect on cell apoptosis in CHF. The underlying mechanism may be attributed to the increased secretion of miR-155 in exosomes and the subsequent inhibition of p38 MAPK protein phosphorylation. Our findings not only provide evidence for the clinical treatment of CHF with WZG, but also offer new insights into the role of exosomal miR-155 in the regulation of cardiomyocyte apoptosis.

## Data Availability

The original contributions presented in the study are included in the article/Supplementary Material, further inquiries can be directed to the corresponding authors.

## References

[B1] Cai H. Z. Chen X. Y. Xu Z. L. Wu Z. Y. (2019). Effects of Wenyangzhenshuai Granule on miR-155/P38MAPK regulates myocardial cell injury model. Lishizhen Med. Mater Med. Res. 30 (02), 257–260. 10.3969/j.issn.1008-0805.2019.02.001

[B2] Chen W. Luo X. Li W. Li X. Wang Y. Zhang R. (2025). Uncovering the active ingredients of Xinbao pill against chronic heart failure: a chemical profiling, pharmacokinetics and pharmacodynamics integrated study. J. Ethnopharmacol. 342, 119418. 10.1016/j.jep.2025.119418 39880064

[B3] Colombo M. Raposo G. Théry C. (2014). Biogenesis, secretion, and intercellular interactions of exosomes and other extracellular vesicles. Annu. Rev. Cell Dev. Biol. 30, 255–289. 10.1146/annurev-cellbio-101512-122326 25288114

[B4] Cunha E. R. K. Ying W. Olefsky J. M. (2024). Exosome-mediated impact on systemic metabolism. Annu. Rev. Physiol. 86, 225–253. 10.1146/annurev-physiol-042222-024535 38345906

[B5] Du S. Ling H. Guo Z. Cao Q. Song C. (2021). Roles of exosomal miRNA in vascular aging. Pharmacol. Res. 165, 105278. 10.1016/j.phrs.2020.105278 33166733

[B6] Eshraghi R. Rafiei M. Hadian Jazi Z. Shafie D. Raisi A. Mirzaei H. (2024). MicroRNA-155 and exosomal microRNA-155: small pieces in the cardiovascular diseases puzzle. Pathol. Res. Pract. 257, 155274. 10.1016/j.prp.2024.155274 38626659

[B7] Ge X. Meng Q. Wei L. Liu J. Li M. Liang X. (2021). Myocardial ischemia-reperfusion induced cardiac extracellular vesicles harbour proinflammatory features and aggravate heart injury. J. Extracell. Vesicles 10 (4), e12072. 10.1002/jev2.12072 33664937 PMC7902529

[B8] Gong X. Y. Zhang Y. (2019). Protective effect of miR-20a against hypoxia/reoxygenation treatment on cardiomyocytes cell viability and cell apoptosis by targeting TLR4 and inhibiting p38 MAPK/JNK signaling. Vitro Cell Dev. Biol. Anim. 55 (10), 793–800. 10.1007/s11626-019-00399-4 31444671

[B9] Guduric-Fuchs J. O'Connor A. Camp B. O'Neill C. L. Medina R. J. Simpson D. A. (2012). Selective extracellular vesicle-mediated export of an overlapping set of microRNAs from multiple cell types. BMC Genomics 13, 357. 10.1186/1471-2164-13-357 22849433 PMC3532190

[B10] Guo J. Yang P. Li Y. F. Tang J. F. He Z. X. Yu S. G. (2023). MicroRNA: crucial modulator in purinergic signalling involved diseases. Purinergic Signal 19 (1), 329–341. 10.1007/s11302-022-09840-y 35106737 PMC9984628

[B11] Gurung S. Perocheau D. Touramanidou L. Baruteau J. (2021). The exosome journey: from biogenesis to uptake and intracellular signalling. Cell Commun. Signal 19 (1), 47. 10.1186/s12964-021-00730-1 33892745 PMC8063428

[B12] Han C. Yang J. Sun J. Qin G. (2022). Extracellular vesicles in cardiovascular disease: biological functions and therapeutic implications. Pharmacol. Ther. 233, 108025. 10.1016/j.pharmthera.2021.108025 34687770 PMC9018895

[B13] He L. Sun G. B. Sun X. Sun J. Sun X. B. (2012). Protective effects of luteolin-7-0-glucoside on neonatal rat cardiomyocytes damage induced by adriamycin. Chin. Pharmacol. Bull. 28 (09), 1229–1234. 10.3969/j.issn.1001-1978.2012.09.011

[B14] He W. Huang H. Xie Q. Wang Z. Fan Y. Kong B. (2016). MiR-155 knockout in fibroblasts improves cardiac remodeling by targeting tumor protein p53-inducible nuclear protein 1. J. Cardiovasc Pharmacol. Ther. 21 (4), 423–435. 10.1177/1074248415616188 26589288

[B15] He X. Liu S. Zhang Z. Liu Q. Dong J. Lin Z. (2024). M1 macrophage-derived exosomes inhibit cardiomyocyte proliferation through delivering miR-155. BMC Cardiovasc Disord. 24 (1), 365. 10.1186/s12872-024-03893-0 39014329 PMC11251235

[B16] Heinrich M. Appendino G. Efferth T. Fürst R. Izzo A. A. Kayser O. (2020). Best practice in research - overcoming common challenges in phytopharmacological research. J. Ethnopharmacol. 246, 112230. 10.1016/j.jep.2019.112230 31526860

[B17] Hu C. Liao J. Huang R. Su Q. He L. (2023). MicroRNA-155-5p in serum derived-exosomes promotes ischaemia-reperfusion injury by reducing CypD ubiquitination by NEDD4. Esc. Heart Fail 10 (2), 1144–1157. 10.1002/ehf2.14279 36631006 PMC10053265

[B18] Jia J. Li X. Guo S. Xie X. (2020). MicroRNA-155 suppresses the translation of p38 and impairs the functioning of dendritic cells in endometrial cancer mice. Cancer Manag. Res. 12, 2993–3002. 10.2147/CMAR.S240926 32431542 PMC7198441

[B19] Jo S. Samarpita S. Lee J. S. Lee Y. J. Son J. E. Jeong M. (2022). 8-Shogaol inhibits rheumatoid arthritis through targeting TAK1. Pharmacol. Res. 178, 106176. 10.1016/j.phrs.2022.106176 35283302

[B20] Kalluri R. LeBleu V. S. (2020). The biology, function, and biomedical applications of exosomes. Science 367 (6478), eaau6977. 10.1126/science.aau6977 32029601 PMC7717626

[B21] Li Z. Ma J. Y. Kerr I. Chakravarty S. Dugar S. Schreiner G. (2006). Selective inhibition of p38alpha MAPK improves cardiac function and reduces myocardial apoptosis in rat model of myocardial injury. Am. J. Physiol. Heart Circ. Physiol. 291 (4), H1972–H1977. 10.1152/ajpheart.00043.2006 16751295

[B22] Li K. Ma L. Lu Z. Yan L. Chen W. Wang B. (2023). Apoptosis and heart failure: the role of non-coding RNAs and exosomal non-coding RNAs. Pathol. Res. Pract. 248, 154669. 10.1016/j.prp.2023.154669 37422971

[B23] Li C. Zhang X. Li J. Liang L. Zeng J. Wen M. (2023). Ginsenoside Rb1 promotes the activation of PPARα pathway via inhibiting FADD to ameliorate heart failure. Eur. J. Pharmacol. 947, 175676. 10.1016/j.ejphar.2023.175676 37001580

[B24] Lin Q. C. Chen X. Y. Dai F. Y. Wen Z. (2019). Clinical research of Wenyang Zhenxi Granules combined with Levosimendan in chronic heart failure patients with Yang deficiency and water flooding syndrome. Guid J. Tradit. Chin. Med. Pharmacol. 25 (06), 82–5+94. 10.13862/j.cnki.cn43-1446/r.2019.06.023

[B25] Liu C. Liu H. Sun Q. Zhang P. (2021). MicroRNA 1283 alleviates cardiomyocyte damage caused by hypoxia/reoxygenation via targeting GADD45A and inactivating the JNK and p38 MAPK signaling pathways. Kardiol. Pol. 79 (2), 147–155. 10.33963/KP.15696 33293495

[B26] Liu M. Fu D. Gao T. Jiang H. Yang P. Li X. (2024). The low expression of miR-155 promotes the expression of SHP2 by inhibiting the activation of the ERK1/2 pathway and improves cell pyroptosis induced by I/R in mice. Aging (Albany NY) 16 (5), 4778–4788. 10.18632/aging.205631 38451182 PMC10968689

[B27] Luo Y. Deng X. Chen Q. Cai Y. Bie M. Zhang Y. (2023). Up-regulation of miR-155 protects against chronic heart failure by inhibiting HIF-1α. Am. J. Transl. Res. 15 (11), 6425–6436. 38074801 PMC10703650

[B28] Ma X. L. Kumar S. Gao F. Louden C. S. Lopez B. L. Christopher T. A. (1999). Inhibition of p38 mitogen-activated protein kinase decreases cardiomyocyte apoptosis and improves cardiac function after myocardial ischemia and reperfusion. Circulation 99 (13), 1685–1691. 10.1161/01.cir.99.13.1685 10190877

[B29] Marber M. S. Rose B. Wang Y. (2011). The p38 mitogen-activated protein kinase pathway--a potential target for intervention in infarction, hypertrophy, and heart failure. J. Mol. Cell Cardiol. 51 (4), 485–490. 10.1016/j.yjmcc.2010.10.021 21062627 PMC3061241

[B30] Martin E. D. Bassi R. Marber M. S. (2015). p38 MAPK in cardioprotection - are we there yet? Br. J. Pharmacol. 172 (8), 2101–2113. 10.1111/bph.12901 25204838 PMC4386984

[B31] Martínez-Limón A. Joaquin M. Caballero M. Posas F. de Nadal E. (2020). The p38 pathway: from biology to cancer therapy. Int. J. Mol. Sci. 21 (6), 1913. 10.3390/ijms21061913 32168915 PMC7139330

[B32] Meiri E. Volinsky N. Dromi N. Kredo-Russo S. Benjamin H. Tabak S. (2020). Differential expression of microRNA in serum fractions and association of Argonaute 1 microRNAs with heart failure. J. Cell Mol. Med. 24 (12), 6586–6595. 10.1111/jcmm.15306 32400052 PMC7299714

[B33] Metra M. Tomasoni D. Adamo M. Bayes-Genis A. Filippatos G. Abdelhamid M. (2023). Worsening of chronic heart failure: definition, epidemiology, management and prevention. A clinical consensus statement by the Heart Failure Association of the European Society of Cardiology. Eur. J. Heart Fail 25 (6), 776–791. 10.1002/ejhf.2874 37208936

[B34] Oy G. Cai H. Z. Wang Y. L. Wang M. Chen Q. Y. Liu Y. M. (2020). Effects of Wenyang Zhenshuai Granules on the expression of p38MAPK in the plasma exosomes and the tissues of heart and kidney of cardio-renal syndrome model rats. J. Tradit. Chin. Med. 61 (15), 1344–1349. 10.13288/j.11-2166/r.2020.15.014

[B35] Oh J. H. Kim G. B. Seok H. (2022). Implication of microRNA as a potential biomarker of myocarditis. Clin. Exp. Pediatr. 65 (5), 230–238. 10.3345/cep.2021.01802 35240034 PMC9082251

[B36] Ou Y. G. Cai H. Z. Wang Y. L. Wang M. Liu Y. M. Chen X. Y. (2019). Exosome miR-155 regulates p38MAPK signaling pathway and affects cardiomyocyt-nephrocyte apoptosis in rats with cardiorenal syndrome. Chin. J. Nephrol. Dial. Transpl. 28 (06), 532–537. 10.3969/j.issn.1006-298X.2019.06.006

[B37] Peng L. Li S. Cai H. Chen X. Tang Y. (2024). Ginsenoside Rg1 treats chronic heart failure by downregulating ERK1/2 protein phosphorylation. Vitro Cell Dev. Biol. Anim. 60 (9), 1085–1098. 10.1007/s11626-024-00960-w 39251466

[B38] Reagan-Shaw S. Nihal M. Ahmad N. (2008). Dose translation from animal to human studies revisited. Faseb J. 22 (3), 659–661. 10.1096/fj.07-9574LSF 17942826

[B39] Roger V. L. (2021). Epidemiology of heart failure: a contemporary perspective. Circ. Res. 128 (10), 1421–1434. 10.1161/CIRCRESAHA.121.318172 33983838

[B40] Romero-Becerra R. Santamans A. M. Folgueira C. Sabio G. (2020). p38 MAPK pathway in the heart: new insights in health and disease. Int. J. Mol. Sci. 21 (19), 7412. 10.3390/ijms21197412 33049962 PMC7582802

[B41] Saliminejad K. Khorram Khorshid H. R. Soleymani Fard S. Ghaffari S. H. (2019). An overview of microRNAs: biology, functions, therapeutics, and analysis methods. J. Cell Physiol. 234 (5), 5451–5465. 10.1002/jcp.27486 30471116

[B42] Santangelo L. Giurato G. Cicchini C. Montaldo C. Mancone C. Tarallo R. (2016). The RNA-binding protein SYNCRIP is a component of the hepatocyte exosomal machinery controlling MicroRNA sorting. Cell Rep. 17 (3), 799–808. 10.1016/j.celrep.2016.09.031 27732855

[B43] Sanz-Ezquerro J. J. Cuenda A. (2021). p38 signalling pathway. Int. J. Mol. Sci. 22 (3), 1003. 10.3390/ijms22031003 33498296 PMC7863928

[B44] Seok H. Y. Chen J. Kataoka M. Huang Z. P. Ding J. Yan J. (2014). Loss of MicroRNA-155 protects the heart from pathological cardiac hypertrophy. Circ. Res. 114 (10), 1585–1595. 10.1161/CIRCRESAHA.114.303784 24657879 PMC4033580

[B45] Shati A. A. (2020). Doxorubicin-induces NFAT/Fas/FasL cardiac apoptosis in rats through activation of calcineurin and P38 MAPK and inhibition of mTOR signalling pathways. Clin. Exp. Pharmacol. Physiol. 47 (4), 660–676. 10.1111/1440-1681.13225 31811646

[B46] Song X. Cui Y. Zhu T. (2023). MicroRNA-19 upregulation attenuates cardiac fibrosis via targeting connective tissue growth factor. Am. J. Med. Sci. 365 (4), 375–385. 10.1016/j.amjms.2022.12.010 36539014

[B47] Sun J. Wang R. Chao T. Peng J. Wang C. Chen K. (2023). Ginsenosi*de re* inhibits myocardial fibrosis by regulating miR-489/myd88/NF-κB pathway. J. Ginseng Res. 47 (2), 218–227. 10.1016/j.jgr.2021.11.009 36926602 PMC10014187

[B48] Sunagawa Y. Funamoto M. Suzuki A. Shimizu K. Sakurai R. Katanasaka Y. (2017). A novel target molecule of nobiletin derived from citrus peels has a therapeutic potency against the development of heart failure. Eur. Cardiol. 12 (2), 105. 10.15420/ecr.2017:23:14 30416575 PMC6213126

[B49] Sunagawa Y. Iwashimizu S. Ono M. Mochizuki S. Iwashita K. Sato R. (2025). The citrus flavonoid nobiletin prevents the development of doxorubicin-induced heart failure by inhibiting apoptosis. J. Pharmacol. Sci. 158 (2), 84–94. 10.1016/j.jphs.2025.03.011 40288827

[B50] The W. (2023). Report on cardiovascular health and diseases in China 2022: an updated summary. Biomed. Environ. Sci. 36 (8), 669–701. 10.3967/bes2023.106 37711081

[B51] Trajkovic K. Hsu C. Chiantia S. Rajendran L. Wenzel D. Wieland F. (2008). Ceramide triggers budding of exosome vesicles into multivesicular endosomes. Science 319 (5867), 1244–1247. 10.1126/science.1153124 18309083

[B52] Villarroya-Beltri C. Gutiérrez-Vázquez C. Sánchez-Cabo F. Pérez-Hernández D. Vázquez J. Martin-Cofreces N. (2013). Sumoylated hnRNPA2B1 controls the sorting of miRNAs into exosomes through binding to specific motifs. Nat. Commun. 4, 2980. 10.1038/ncomms3980 24356509 PMC3905700

[B53] Virani S. S. Alonso A. Aparicio H. J. Benjamin E. J. Bittencourt M. S. Callaway C. W. (2021). Heart disease and stroke statistics-2021 update: a report from the American heart association. Circulation 143 (8), e254–e743. 10.1161/CIR.0000000000000950 33501848 PMC13036842

[B54] Wang Y. Huang S. Sah V. P. Ross J. Jr. Brown J. H. Han J. (1998). Cardiac muscle cell hypertrophy and apoptosis induced by distinct members of the p38 mitogen-activated protein kinase family. J. Biol. Chem. 273 (4), 2161–2168. 10.1074/jbc.273.4.2161 9442057

[B55] Wang H. Bei Y. Huang P. Zhou Q. Shi J. Sun Q. (2016). Inhibition of miR-155 protects against LPS-induced cardiac dysfunction and apoptosis in mice. Mol. Ther. Nucleic Acids 5 (10), e374. 10.1038/mtna.2016.80 27727247 PMC5095684

[B56] Xiao Y. C. Wang W. Gao Y. Li W. Y. Tan X. Wang Y. K. (2022). The peripheral circulating exosomal microRNAs related to central inflammation in chronic heart failure. J. Cardiovasc Transl. Res. 15 (3), 500–513. 10.1007/s12265-022-10266-5 35501543

[B57] Xu Z. L. Chen X. Y. Chen Q. Y. Wu Z. Y. Cai H. Z. (2020). Effects of Wenyangzhenshuai granule on LncRNA BIC/miR-155 regulating P38MAPK expression in chronic heart failure rats. Chin. J. Gerontol. 40 (16), 3496–3499. 10.3969/j.issn.1005-9202.2020.16.043

[B58] Xu Y. Zhang C. Cai D. Zhu R. Cao Y. (2023). Exosomal miR-155-5p drives widespread macrophage M1 polarization in hypervirulent Klebsiella pneumoniae-induced acute lung injury via the MSK1/p38-MAPK axis. Cell Mol. Biol. Lett. 28 (1), 92. 10.1186/s11658-023-00505-1 37953267 PMC10641976

[B59] Xu S. Hu C. Han J. Luo W. Huang L. Jiang Y. (2024). Schisandrin B alleviates angiotensin II-induced cardiac inflammatory remodeling by inhibiting the recruitment of MyD88 to TLRs in mouse cardiomyocytes. Int. Immunopharmacol. 139, 112660. 10.1016/j.intimp.2024.112660 39018688

[B60] Xue R. Tan W. Wu Y. Dong B. Xie Z. Huang P. (2020). Role of exosomal miRNAs in heart failure. Front. Cardiovasc Med. 7, 592412. 10.3389/fcvm.2020.592412 33392270 PMC7773699

[B61] Yan F. Cui W. Chen Z. (2022). Mesenchymal stem cell-derived exosome-loaded microRNA-129-5p inhibits TRAF3 expression to alleviate apoptosis and oxidative stress in heart failure. Cardiovasc Toxicol. 22 (7), 631–645. 10.1007/s12012-022-09743-9 35546649

[B62] Yang L. Zhang M. H. Cai H. Z. Shi J. L. (2017). Content determination of ginsenoside Rg1, Ginsenoside Re and ginsenoside Rb1 in Wenyang Zhenshuai Granules by HPLC. Chin. J. Inf. Tradit. Chin. Med. 24 (04), 75–78. 10.3969/j.issn.1005-5304.2017.04.019

[B63] Yao K. P. Liu Q. L. Shi J. Cai Z. J. Cai H. Z. Chen X. Y. (2021). Mechanism of Wenyang Zhenshuai granule in treatment of chronic heart failure based on network pharmacology and molecular docking. J. Hunan Univ. Chin. Med. 41 (09), 1365–1371. 10.3969/j.issn.1674-070X.2021.09.010

[B64] You G. Chen X. Ma X. Tang X. Zheng Y. Lin W. (2025). TAT and RVG Co-modified MSC-derived exosomes-mediated delivery of microRNA-15b-5p inhibitor alleviate cerebral ischemia and reperfusion-induced neuronal apoptosis by promoting HTR2C-ERK signaling. Mol. Neurobiol. 10.1007/s12035-025-05249-x 40711710

[B65] Yu H. Qin L. Peng Y. Bai W. Wang Z. (2020). Exosomes derived from hypertrophic cardiomyocytes induce inflammation in macrophages via miR-155 mediated MAPK pathway. Front. Immunol. 11, 606045. 10.3389/fimmu.2020.606045 33613526 PMC7886800

[B66] Yuan Y. Mei Z. Qu Z. Li G. Yu S. Liu Y. (2023). Exosomes secreted from cardiomyocytes suppress the sensitivity of tumor ferroptosis in ischemic heart failure. Signal Transduct. Target Ther. 8 (1), 121. 10.1038/s41392-023-01336-4 36967385 PMC10040407

[B67] Zheng D. Huo M. Li B. Wang W. Piao H. Wang Y. (2020). The role of exosomes and exosomal MicroRNA in cardiovascular disease. Front. Cell Dev. Biol. 8, 616161. 10.3389/fcell.2020.616161 33511124 PMC7835482

