# Supplementary material for: Wenyang Zhenshuai Granules inhibits cardiomyocyte apoptosis in chronic heart failure by regulating p38 MAPK signaling pathway through exosomal miR-155
Source: Front Pharmacol. 2025 Oct 3;16:1538091. doi: 10.3389/fphar.2025.1538091 (PMC12531156; doi:10.3389/fphar.2025.1538091)
Supplement: Supplementary file 1 [file DataSheet1.pdf]

Supplementary materials for Western blotting

Fig 3(B)

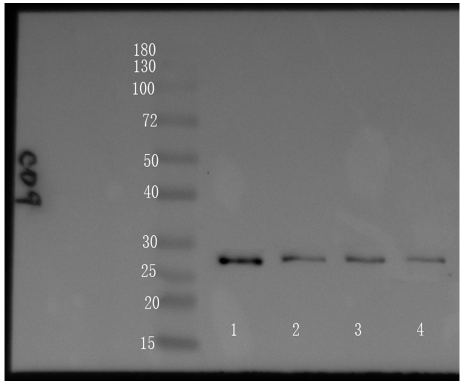

CD9

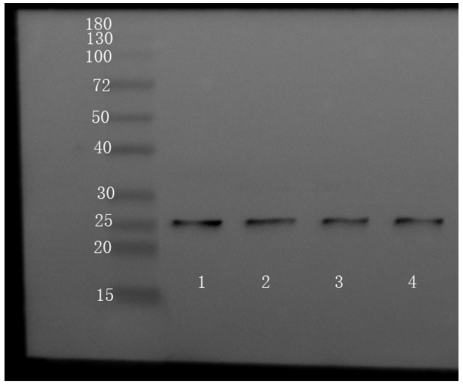

CD63

Fig 4(A)

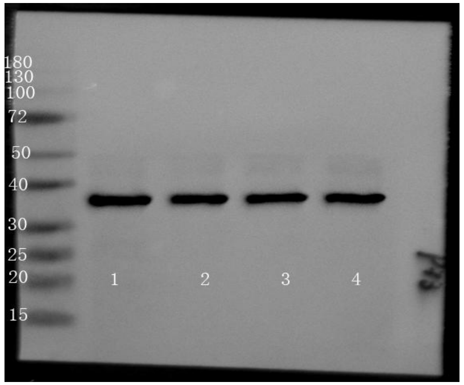

p38 MAPK

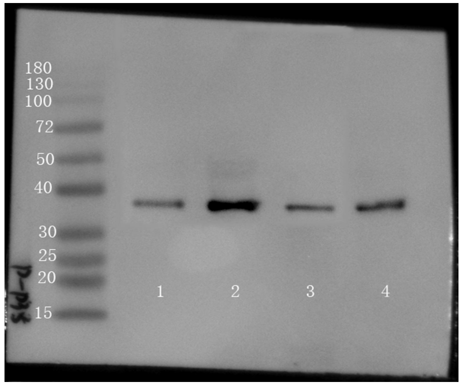

p-p38 MAPK

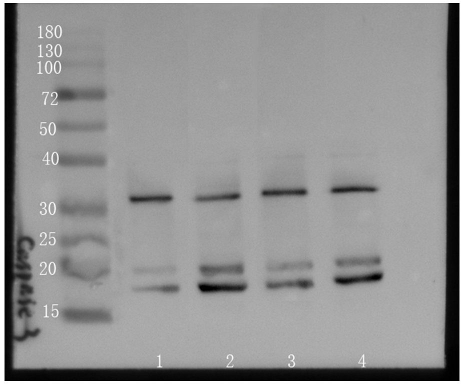

Caspase-3

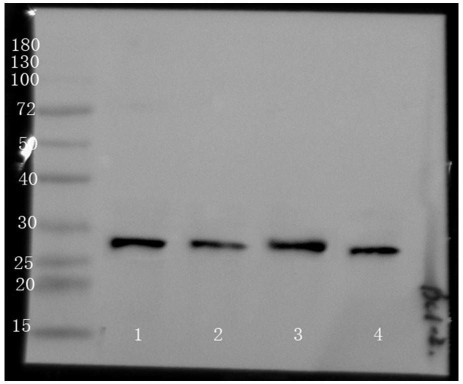

Bcl-2

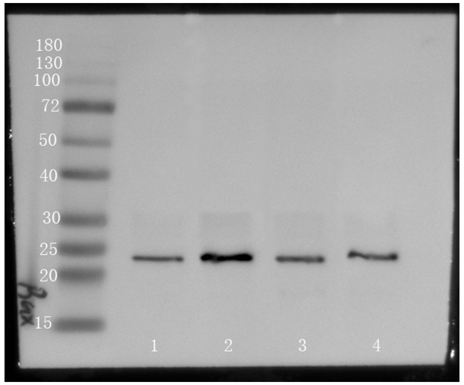

Bax

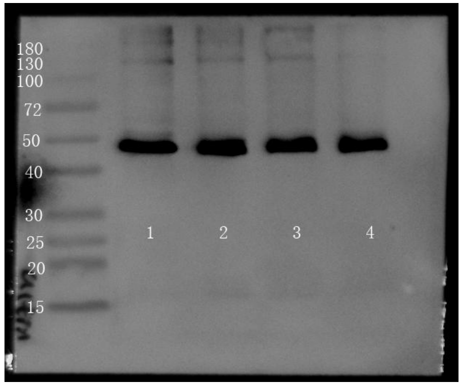

***β*-actin**
